# Microbial Infection and Antibiotic Susceptibility of Diabetic Foot Ulcer in China: Literature Review

**DOI:** 10.3389/fendo.2022.881659

**Published:** 2022-05-19

**Authors:** Fang Du, Jing Ma, Hongping Gong, Raju Bista, Panpan Zha, Yan Ren, Yun Gao, Dawei Chen, Xingwu Ran, Chun Wang

**Affiliations:** Diabetic Foot Care Center, Department of Endocrinology and Metabolism, West China Hospital, Sichuan University, Chengdu, China

**Keywords:** microbial infection, antibiotic susceptibility, diabetic foot ulcer, diabetic foot infection, multi-drug resistant organisms, polymicrobial infection

## Abstract

**Objective:**

To investigate the microbial spectrum isolated from foot ulcers among diabetic patients in China, which was conducted to help clinicians choose optimal antibiotics empirically.

**Method:**

The PubMed, MEDLINE, Web of Science, China Biology Medicine (CBM), China National Knowledge Infrastructure (CNKI), WanFang, and VIP databases were searched for studies published between 2015 to 2019, that report primary data on diabetic foot infection (DFI) and antibiotic susceptibility in China.

**Result:**

A total of 63 articles about DFI and antibiotic susceptibility tests among diabetic patients in China were included. There were 11,483 patients with an average age of 60.2 ± 10.1 years and a mean course of 10.6 ± 5.0 years between 2010 and 2019, covering most geographical regions of China. The prevalence of Gram-positive (GP) bacteria (43.4%) was lower than that of Gram-negative (GN) (52.4%). The most prevalent pathogens isolated were Staphylococcus aureus (17.7%), Escherichia coli (10.9%), Pseudomonas aeruginosa (10.5%), Klebsiella pneumoniae (6.2%), Staphylococcus epidermidis (5.3%), Enterococcus faecalis (4.9%), and fungus (3.7%). The prevalence of polymicrobial infection was 22.8%. GP bacteria were sensitive to linezolid, vancomycin, and teicoplanin. More than 50% of GN bacteria were resistant to third-generation cephalosporins, while the resistance rates of piperacillin/tazobactam, amikacin, meropenem, and imipenem were relatively low. Among the 6017 strains of the isolated organisms, 20% had multi-drug resistance (MDR). Staphylococcus aureus (30.4%) was the most predominant MDR bacteria, followed by extended-spectrum β-lactamase (ESBL) (19.1%).

**Conclusion:**

The microbial infection of foot ulcers among diabetic patients in China is diverse. The microbial spectrum is different in different geographic regions and Staphylococcus aureus is the predominant bacteria. Polymicrobial and MDR bacterial infections on the foot ulcers are common. This study could be valuable in guiding the empirical use of antibiotics for diabetic foot infections.

## Introduction

The prevalence of global diabetic foot ulcers (DFU) is about 6.3% (5.4-7.3%) (1). It is one of the most serious and costly complications of diabetes. In total, 25% of diabetic patients develop a foot ulcer in their lifetime. It has been predicted that nearly 50% of patients with DFU suffer from foot infections (2). About 20% of moderate or severe diabetic foot infections (DFI) lead to minor or major amputation (3). DFI is a risk factor for poor wound healing, amputation, and premature mortality.

DFI usually begins with a break in the protective cutaneous envelope, typically in a site of trauma or ulceration, most often in a patient with peripheral neuropathy and frequently with peripheral artery disease (4). If the infection is not detected early and controlled timely, it can spread from the superficial tissue to the deep structures such as bone and joints. Antibiotics are indispensable for the treatment of DFI. Clinicians mostly have to use initial antibiotics empirically before the result of microbial culture is available. False diagnosis of DFI leads to unnecessary overuse or misuse of antibiotics. Furthermore, the types of pathogens and drug resistance rate of DFI are rising dramatically, due to the widespread use of broad-spectrum antibiotics and variations in antibiotic resistance genes (5, 6). Antibiotic therapy in the past may have influenced the bacterial spectrum of foot ulcers. The pathogenic organisms on the DFU, on the other hand, vary and are linked to location, economy, environment, lifestyle, and awareness. As a consequence, when treating DFI it is important that clinicians carefully select appropriate antibiotics.

This study reviews literature on DFI published between 2015 and 2019. It then synthesizes multiple data, summarizing the microbial distribution of DFU in China before analyzing antibiotic sensitivity to prompt initiation of optimal antimicrobial therapy.

## Methods

### Literature Search Strategy

China Biology Medicine (CBM), China National Knowledge Infrastructure (CNKI), WanFang, VIP, PubMed, MEDLINE, and Web of Science databases were electronically searched to collect studies about DFI and antibiotic sensitivity tests, published from January 2015 to December 2019. We used the following search terms: diabetic foot, diabetic foot ulcer, foot ulcer, infection, microbiology, bacteria, fungus, mycoses, anti-infective agents, drug(antibiotic) sensitivity, drug(antibiotic) sensitivity test, drug resistance, and antibiotic resistance.

### Study Inclusion Criteria

Relevant studies were selected based on the following criteria: a clinical study of bacterial infection and a drug sensitivity test in diabetic foot ulcers. We excluded case reports, studies without antibiotic sensitivity, studies on *in vitro* experiments, articles on the topical treatment of diabetic foot ulcers, and articles on specific pathogens. Articles that were not Chinese scientific and technological papers statistical source journals and tertiary hospitals were also excluded.

### Data Abstraction and Quality Appraisal

The following data were abstracted onto standardized forms: publication year, first author, research area, study design, research time, patients’ number, age and gender of patients, duration of diabetes, number and distribution of pathogenic bacteria, results of drug sensitive tests. Data extraction and article quality assessment were carried out independently by two researchers.

### Statistical Analysis

We used SPSS version 20.0 statistical software to analyze the data. Qualitative variables were described using percentages. Quantitative variables conforming to normal distribution were described by mean ± SD, and quantitative variables not conforming to normal distribution were described by Median (minimum-maximum). Statistical significance was considered at a p value <0.05.

## Results

### Clinical Characteristics of the Patients With DFU

**Figure 1:**
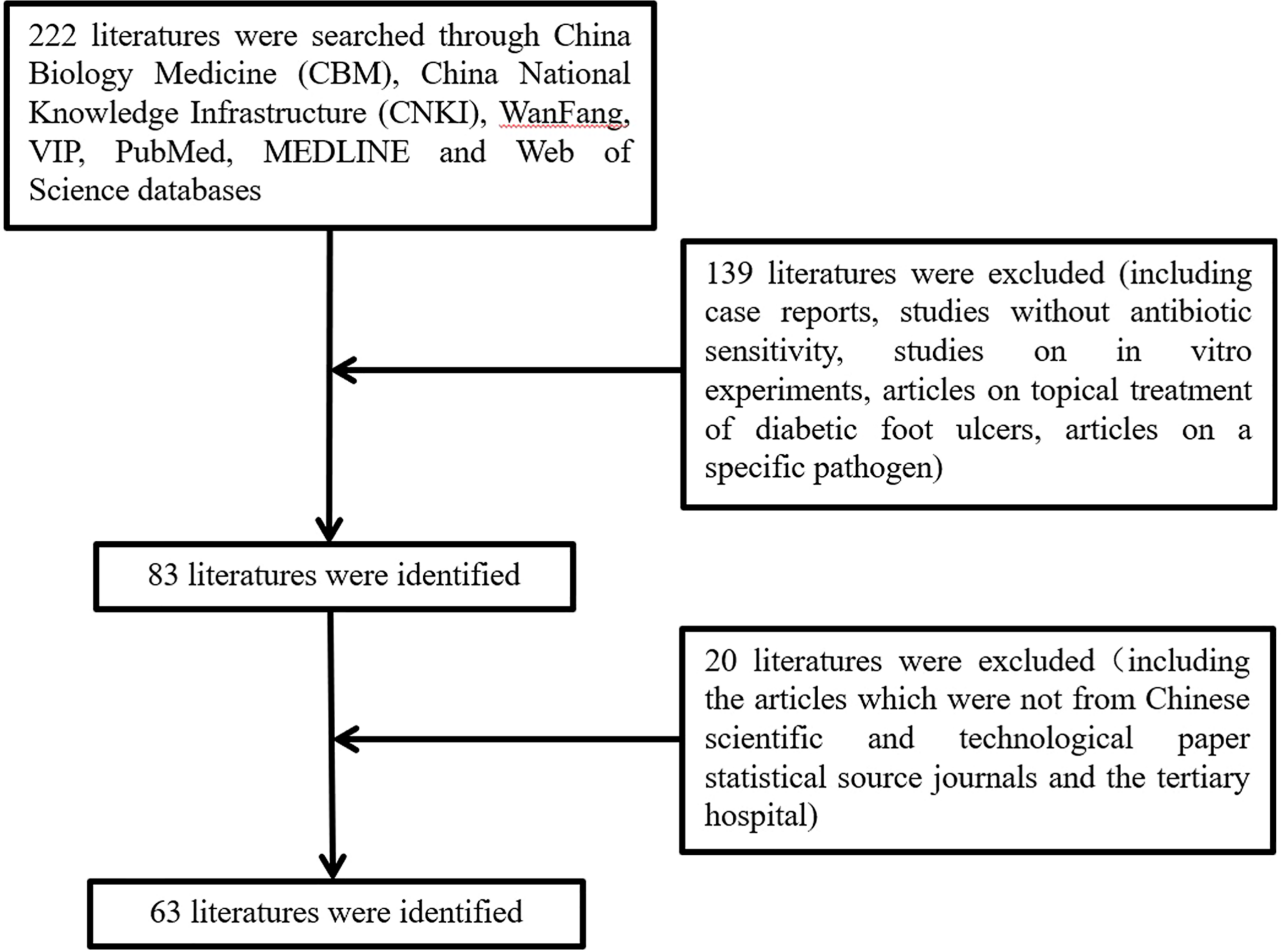
Flow chart of literature collecting.

In total, the study included 63 articles published between 2015 and 2019 (**Figure 1** and **Supplementary Material**), which were retrospective clinical studies, involving 11483 DFU patients admitted to tertiary hospitals in China from January 2010 to September 2019 and covering most geographical regions of China, including Northeast (provinces of Heilongjiang, Jilin, and Liaoning), North China (Beijing and Tianjin cities, province of Hebei), Central China (provinces of Henan, Hunan, and Hubei), Southern China (provinces of Hainan and Guangdong, Guangxi Zhuang Autonomous Region), East China (provinces of Jiangsu, Zhejiang, Anhui, Fujian, and Shandong), Northwest (Xinjiang Uygur Autonomous Region) and Southwest (provinces of Guizhou, Sichuan, and Yunnan, Chongqing city). Overall, the average age of the DFU patients was 60.2 ± 10.1 years (18-97 yr), and 61.0% of the patients were male. The mean duration of diabetes was 10.6 ± 5.0 years.

### Distribution of Pathogens

A total of 12292 strains of pathogenic bacteria were isolated, including 5335 (43.4%) strains of gram-positive (GP) organisms, 6441 (52.4%) gram-negative (GN) bacilli, and 459 (3.7%) fungal strains. Staphylococcus aureus, Escherichia coli, and Pseudomonas aeruginosa were the most frequently isolated from the foot ulcers, which accounted for more than 10% (**Table 1**). Staphylococcus aureus, Staphylococcus epidermidis, and Enterococcus faecalis were the main GP bacteria, while Escherichia coli, Pseudomonas aeruginosa, and Klebsiella pneumonia were the main GN bacteria (**Table 1**). Among the isolated fungi, Candida albicans was the most abundant, accounting for about 55.6%.

**Table 1:** Distribution of the pathogenic bacteria isolated from the DFUs.

Organisms	Number of pathogens (n)	Percentage (%)
**Gram-positive bacteria**	5335	43.4
Staphylococcus aureus	2171	17.7
Staphylococcus epidermidis	655	5.3
Enterococcus faecalis	598	4.9
Streptococcus	554	4.5
Staphylococcus haemolyticus	205	1.7
others	1152	9.4
**Gram-negative bacteria**	6441	52.4
Escherichia coli	1335	10.9
Pseudomonas aeruginosa	1290	10.5
Klebsiella pneumonia	763	6.2
Proteus mirabilis	382	3.1
Acinetobacter baumannii	315	2.6
others	2356	19.2
**Fungus**	459	3.7
**Others**	57	0.5

**Figure 2:**
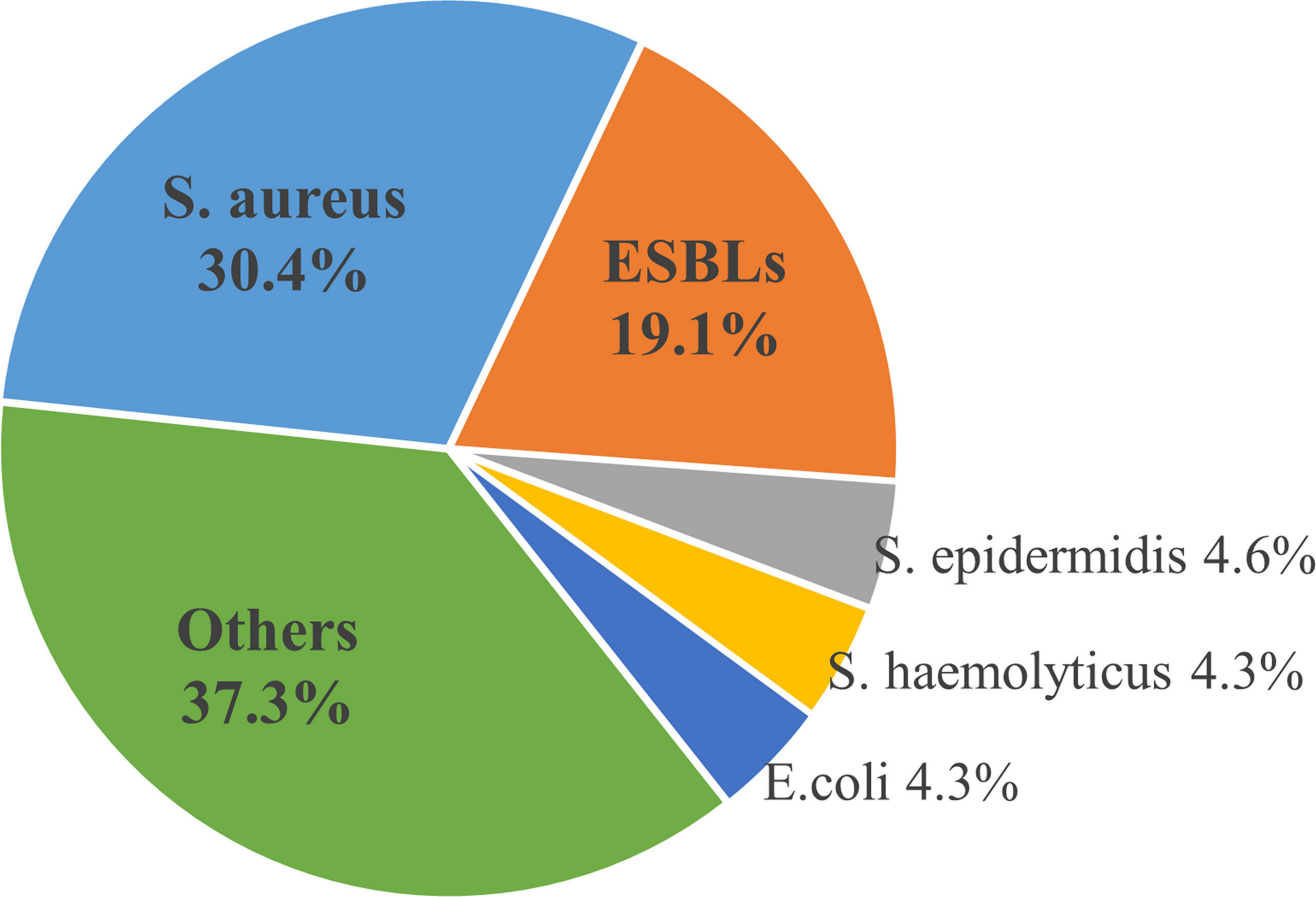
Distribution of common Multiple Drug Resistant Organisms.

There were 1696 (22.8%) cases of multiple-pathogen infections among 7449 patients. And a total of 1217(20%) strains of multidrug-resistant organisms (MDRO) were found among 6071 strains of pathogens in 28 studies, of which 1098 strains of MDRO were described in detail. Staphylococcus aureus was the most common multi-drug resistant bacteria, followed by ESBL, accounting for 30.4% and 19.1%, respectively (**Figure 2**).

### Distribution of Pathogens in Different Geographical Regions of China

**Figure 3:**
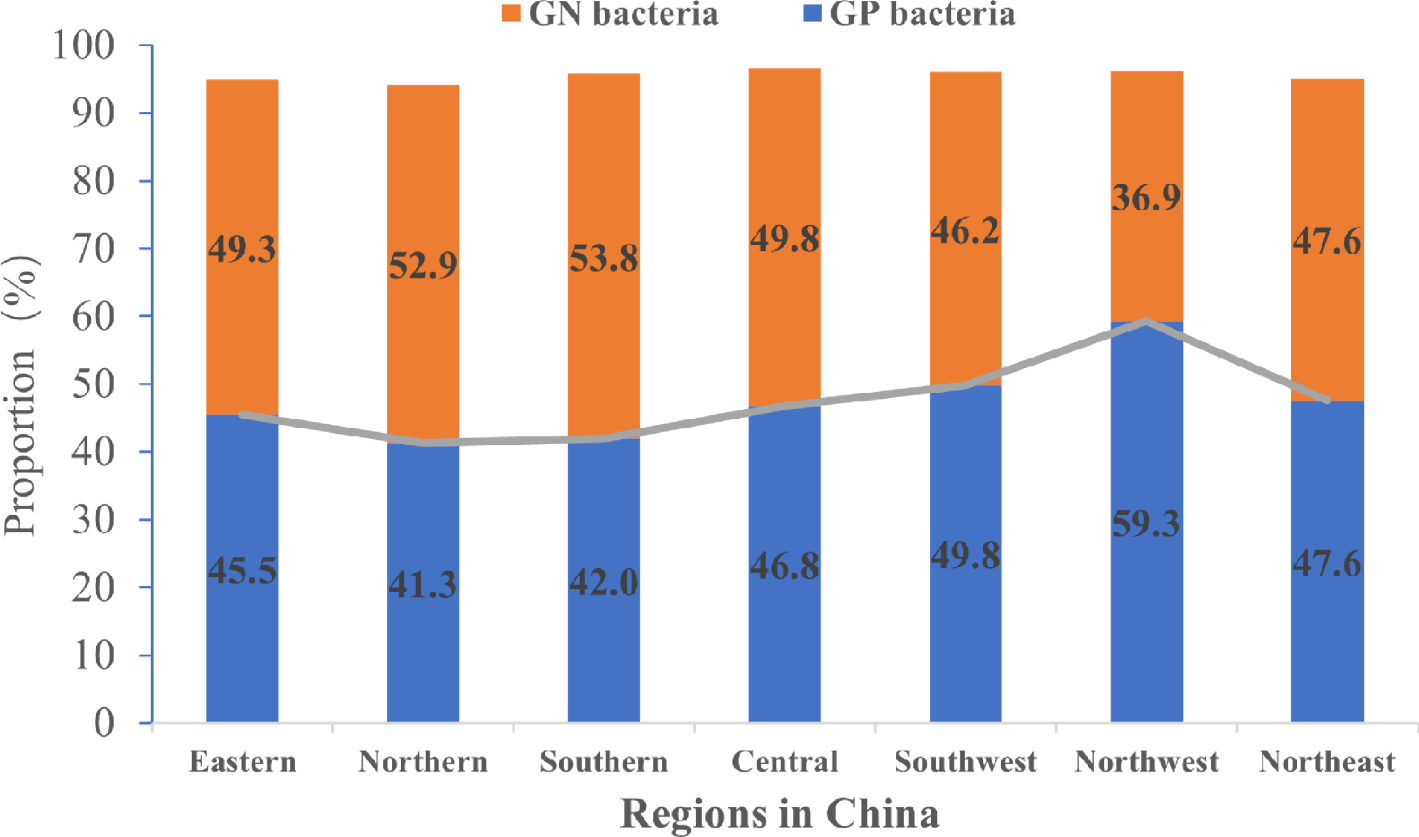
The proportions of GN and GP bacteria in different regions of China.

**Figure 4:**
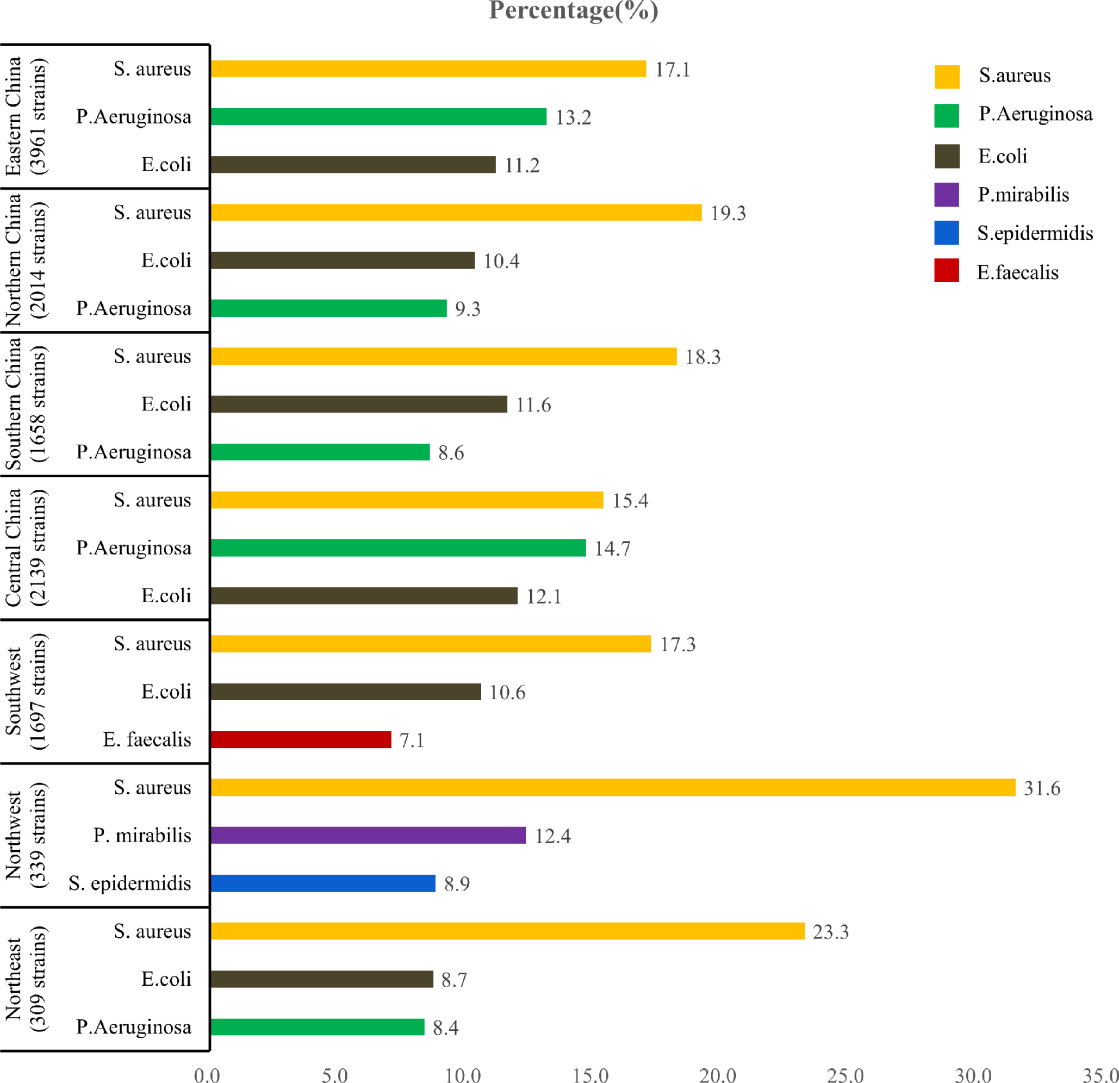
Number of pathogens and main pathogens in different geographical regions of China.

Although, the proportions of GN bacteria and GP bacteria in China were not significantly different (P>0.05), the former was slightly higher than that of the latter (**Figure 3**). Of the cultured bacteria, Staphylococcus aureus was the most common among the patients with DFU in the seven geographic regions of China (**Figure 4**).

### Analysis of Antibiotic Resistance of Pathogenic Bacteria

The resistance rate of GP bacteria to vancomycin, linezolid, and teicoplanin was lower, and to penicillin, penicillin G, oxacillin, ampicillin, cefazolin, cefoxitin, erythromycin, clindamycin, gentamycin, Ciprofloxacin, levofloxacin, tetracycline was higher (**Table 2**). GN bacteria to Piperacillin/tazobactam, Amikacin, meropenem, and imipenem were less resistant, while cephalosporins, including the third-generation cephalosporins were more resistant. In addition, the resistance rate of Escherichia coli to cefotetan was only 9.65% (**Table 3**). The drug susceptibility tests of common bacteria showed that a few strains were resistant to sensitive antibiotics (**Table 4**).

**Table 2:** Antibiotic-resistant rate of common GP bacteria in the drug susceptibility tests (%).

Antibiotic	Staphylococcus aureus	Staphylococcus epidermidis	Enterococcus faecalis
Penicillin	92.2 (34.2-100.0)	93.8 (25.0-100.0)	25.0 (0.0-100.0)
Penicillin G	90.5 (78.6-100.0)	100.0 (28.6-100.0)	57.1 (3.3-100.0)
Oxacillin	39.0 (0.0-100.0)	76.9 (12.5-100.0)	89.2 (80.0-100.0)
Ampicillin	87.5 (57.9-100.0)	88.6 (66.7-100.0)	13.6 (0.0-83.3)
Cefazolin	52.1 (0.0-100.0)	65.2 (14.3-100.0)	37.5 (0.0-100.0)
Cefoxitin	36.4 (0.0-60.0)	39.3 (35.6-50.0)	
Erythromycin	67.6 (28.6-100.0)	78.2 (28.6-100.0)	73.2 (0.0-100.0)
Azithromycin	85.0 (25.0-100.0)		
Clindamycin	59.6 (7.5-100.0)	58.5 (14.3-100.0)	85.7 (14.3-100.0)
Gentamicin	38.5 (0.0-93.6)	33.3 (0.0-84.7)	48.7 (12.5-100.0)
Ciprofloxacin	50.0 (4.6-92.9)	59.2 (22.4-81.9)	42.9 (0.0-100.0)
Levofloxacin	31.4 (0.0-93.3)	59.4 (20.8-100.0)	30.8 (0.0-90.9)
Moxifloxacin	5.4 (0.0-45.9)	20.8 (0.0-100.0)	25.0 (13.3-90.9)
Tetracycline	40.4 (9.1-89.0)	33.3 (0.0-100.0)	69.1 (50.0-100.0)
Vancomycin	0.0 (0.0-14.3)	0.0 (0.0-50.0)	0.0 (0.0-37.5)
Linezolid	0.0 (0.0-15.0)	0.0 (0.0-14.3)	0.0 (0.0-14.3)
Teicoplanin	0.0 (0.0-55.0)	0.0 (0.0-14.30)	6.3 (0.0-33.3)
Rifampicin	5.6 (0.0-95.0)	3.6 (0.0-100.0)	74.3 (33.3-88.9)
Cotrimoxazole	21.7 (3.3-75.0)	44.1 (12.5-78.2)	

Antibiotic-resistant rate: Median (minimum-maximum)

**Table 3:** Antibiotic-resistant rate of common GN bacteria in the drug susceptibility tests (%).

Antibiotic	Escherichia coli	Pseudomonas aeruginosa	Klebsiella pneumoniae
Cefazolin	74.2 (27.2-100.0)	93.5 (2.5-100.0)	63.6 (20.0-100.0)
Cefuroxime	59.2 (15.3-100.0)	88.9 (50.0-100.0)	53.6 (26.8-72.7)
Cefoxitin	22.2 (10.0-60.0)	75.0 (8.7-100.0)	54.6 (14.3-71.4)
Cefotaxime	56.7 (20.0-80.0)	66.7 (25.0-80.0)	57.1 (0.0-100.0)
Ceftizoxime	66.7 (30.0-83.3)	62.5 (16.7-85.7)	46.5 (25.0-71.4)
Cefotetan	9.7 (0.0-66.7)		
Ceftazidime	43.5 (0.0-91.7)	33.9 (0.0-88.8)	35.6 (0.0-80.0)
Cefatriaxone	52.3 (16.7-100.0)	68.7 (16.7-100.0)	41.4 (0.0-100.0)
Cefoperazone	40.0 (0.0-83.3)	62.5 (37.5-100.0)	56.3 (8.4-75.0)
Cefoperazone and sulbactam	20.0 (0.0-86.7)	30.4 (0.0-70.0)	41.5 (0.0-64.3)
Cefepime	40.9 (0.0-91.7)	20.0 (0.0-75.0)	15.5 (0.0-60.0)
Ampicillin	86.7 (16.7-100.0)	100.0 (13.1-100.0)	94.0 (40.0-100.0)
Ampicillin/sulbactam	75.0 (60.0-100.0)	93.6 (57.1-100.0)	33.3 (18.2-54.5)
Piperacillin	32.8 (0.0-100.0)	23.1 (0.0-100.0)	42.9 (0.0-100.0)
Piperacillin/tazobactam	11.6 (0.0-50.0)	15.4 (0.0-62.5)	13.0 (0.0-50.0)
Gentamicin	52.3 (6.7-100.0)	45.2 (0.0-75.0)	40.0 (0.0-79.1)
Amikacin	11.2 (0.0-100.0)	13.5 (0.0-100.0)	13.0 (0.0-66.7)
Ciprofloxacin	66.7 (12.9-91.7)	37.5 (0.0-91.9)	40.0 (10.0-72.7)
Levofloxacin	53.3 (13.3-91.3)	28.4 (0.0-86.5)	25.0 (0.0-72.7)
Imipenem	0.0 (0.0-33.3)	14.7 (0.0-56.3)	0.0 (0.0-42.9)
Meropenem	0.0 (0.0-60.0)	17.5 (0.0-50.0)	9.1 (0.0-100.0)
Tobramycin	46.7 (3.0-75.0)	27.5 (0.0-48.2)	26.6 (0.0-46.4)
Aztreonam	47.4 (6.7-91.7)	45.2 (0.0-69.6)	28.6 (0.0-57.1)
Furantoin	18.2 (0.0-82.6)	67.6 (26.3-97.4)	
Cotrimoxazole	68.7 (14.3-100.0)	94.5 (25.0-100.0)	45.5 (10.0-100.0)

Antibiotic-resistant rate: Median (minimum-maximum).

**Table 4:** Resistance analysis of common bacteria to sensitive antibiotics.

Bacteria	Antibiotic	Sensitive to antibiotics		Resistant to antibiotics	
		Number of literatures (n)	Number of strains(n)	Number of literatures (n)	Number of strains(n)
S. aureus	Mancomycin	47	1850	4	7
	Linezolid	25	1254	4	7
	Teicoplanin	18	673	4	22
S. epidermidis	Vancomycin	21	455	5	7
	Linezolid	15	394	1	2
	Teicoplanin	7	250	3	17
E. faecalis	Vancomycin	27	342	5	12
	Linezolid	15	233	4	7
E.coli	Imipenem	43	997	13	21
	Meropenem	24	607	6	18
K.peneumoniae	Imipenem	28	364	11	20

## Discussion

The majority of the patients with DFU were elderly with an average age of 60.2 ± 10.1 years and long-term diabetes. A retrospective study in Liverpool showed that increasing age predicted a shorter survival time for diabetic patients with new foot ulcers (7). A prospective cohort study showed that the hazard of DFU increased with a longer duration of diabetes (8). Interestingly, more than 60% of diabetic patients with foot ulcers were men. As current studies (9–12), more men than women developed DFU, which could be explained by more outdoor work, poor compliance with foot care, and gender-related differences in lifestyles.

The prevalence of pathogens on foot ulcers is variable in the patients with DFU. Previous studies have reported that GP bacteria were more dominant than the GN in DFI (13–15). A survey performed in Southern China from 2009 to 2014 showed that GP and GN bacterial infections were 54% and 48.8%, respectively (16). However, in this study, GN bacteria replaced GP bacteria as the main pathogens not only in Southern China but also in Northern, Eastern, and Central China. A changing trend in the infective organisms causing DFI with GN bacteria instead of GP bacteria as the commonest microbes could also be found in some developing countries (9). In a study in a tertiary care hospital in Pakistan, GN isolates were 76.27% in DFUs (9). In Turkey, the prospective Turk-DAY trial showed that GN bacteria constituted 60.2% of all the isolates (17). GN bacilli were isolated from DFU more frequently (56.1%) than GP cocci (43.9%) in Egyptian (18). The bacterial nature of DFU infection is related to the duration of the ulcer and previous antibiotic exposure (19). Poor hygiene, delayed diagnosis and treatment of DFI, and inappropriate use of empirical antibiotics (9) may contribute to changes in the antibacterial spectrum. Furthermore, climate differences may influence the infected bacterial spectrum on the foot ulcers, which explains the geographic distinction between bacterial infections. With the increase in temperature and humidity, the proportion of GN bacteria on DFU increased (20). We also noted that proportions of GP and GN bacteria were similar in Northeast China and GP bacteria was predominant in Western China (Northwest and southwest). In a recent study (20), the majority (61.5%) of DFIs were caused by GP germs in German. Therefore, different geographical regions had a different distribution of bacteria on the DFUs, and could be associated with socioeconomic level, climatic conditions, hygiene, and use of footwear.

Even with an increasing proportion of GN bacteria on the DFUs, Staphylococcus aureus was still the most commonly isolated bacteria (17.7%) in China. The prevalence of Staphylococcus aureus may be higher in Mexica (42%) (21), Australia (71.8%) (22), but lower in Turkey (11.4%) (17). Staphylococcus aureus was more frequently isolated from DFU in many countries. Staphylococcus aureus is prone to colonize the skin or mucosal surfaces of diabetic patients, which can produce a wide variety of enzymes and toxins such as protease, lipases, nucleases, hyaluronidases, haemolysins (alpha, beta, gamma, and delta), and collagenase which make host tissues favorable for bacterial growth and tissue invasion (23). The antibiotic susceptibility tests showed that GP bacteria were highly sensitive to vancomycin, linezolid, and teicoplanin with low resistance. However, the results of antibiotic susceptibility tests in different countries were inconsistent. In India, a study (10) showed that all of the GP aerobic bacteria were sensitive to doxycycline. In Kuwait (24), vancomycin was the most effective treatment for GP bacteria.

On the other hand, Escherichia coli and Pseudomonas aeruginosa were the most common GN bacteria (more than 10%) in our study. In a study in Guyana, Pseudomonas aeruginosa (18.8%) was the most common isolate of GN bacteria on the DFU, followed by Escherichia coli (13.9%) (25). In Pakistan (9) and India (10), Escherichia coli (15.7%) and Pseudomonas aeruginosa (24%) were the most frequent bacteria among GN isolates, respectively. However, it was reported that Proteus spp. was more common than Escherichia coli and pseudomonas aeruginosa in the DFUs (26). We found that GN bacteria were more susceptible to imipenem, meropenem, piperacillin/tazobactam, and amikacin. In India, GN isolates, except for Acinetobacter, were highly sensitive to amikacin, cefoperazone/sulbactam, and meropenem (10). In Kuwait, imipenem, piperacillin-tazobactam, and amikacin were the most effective treatments for the GN bacteria (24). In the USA (27) and Iran (28), studies showed that GN bacteria have good sensitivity to amikacin, cefoperazone/sulbactam, meropenem, piperacillin/tazobactam (except for Acinetobacter spp.).

Antibiotic therapy for DFUs is often taken for granted but with the increasing use of broad-spectrum antibiotics, more strains of pathogenic bacteria have become resistant to multiple antibiotics, which are the main source of hospital-acquired infections. This study showed that MDRO accounted for about one-fifth of the isolated pathogens. Furthermore, about one-third of MDRO was S. aureus. A meta-analysis (29) showed that the prevalence of methicillin-resistant Staphylococcus aureus (MRSA) colonization among diabetic patients was 4.75% greater than the non-diabetics. However, more recent studies suggested that the prevalence of MRSA might be decreasing in most countries (30). Lately, the antibiotic resistance problem of greatest concern centered around GN organisms that produce extended-spectrum β-lactamases or carbapenemases (30). The emergence of superbugs represents a more serious threat, as they are resistant to all available antibiotics (31, 32). Biofilm formation on the DFUs plays an important role in the development of antibiotic resistance (33). Therefore, debridement is important and effective to remove the biofilm for the treatment of DFU. Recurrent autologous platelet rich gel (APG) (32), negative pressure wound therapy (NPWT) (32), and S. nux-vomica–ZnO nanocomposite (34) may be effective treatments for MDR bacteria in patients with DFU.

In the study, the proportion of fungi isolated was about 3.7%, of which more than half were Candida albicans. However, the prevalence rate and outcomes of true fungal infections in DFUs are elusive. The prevalence of Candida infection on DFUs was 7% in Kuwait (35) and 4.3% in Croatia (36). More recently, a study in the USA (37) investigated 100 nonhealing DFUs with high-throughput sequencing of the pan-fungal internal transcribed spacer 1 locus, estimating that up to 80% of wounds contain fungi, whereas cultures performed in parallel captured only 5% of colonized wounds. The two most abundant species were Cladosporidium herbarum (41%) and Candida albicans (22%). In light of these new findings, the importance of fungal infection in DFUs merits further appreciation.

It is worth noting that more than 20% of patients with DFUs had a polymicrobial infection, consisting of GP, GN aerobic bacteria and fungus, and even anaerobic bacteria. Multiple species on the DFUs could affect wound healing with a great variety of 28%-66% (38, 39). Usually, moderate and severe DFU in the long term was infected by polymicrobial organisms, whereas the monomicrobe was usually isolated from the early, shallow, and mild DFU. The interaction between microbes within the polymicrobial environments led to the production of virulence factors, which can cause inflammation, hinder wound healing, and contribute to the chronicity of the infections (40, 41). Thus, polymicrobial infection of the DFUs could result in treatment failures (42). Identification of pathogenic microbes and differentiation of colonizing bacteria are important for the choice of antibiotics. Empirical antibiotic regimens for the treatment of DFI for the DFU patients with risk of polymicrobial infection include ampicillin/sulbactam, ceftriaxone plus clindamycin or metronidazole, levofloxacin plus clindamycin, moxifloxacin and ertapenem (43). Once multiple organisms are cultivated and sensitivity results are obtained, antibiotics should be adjusted accordingly.

These data are limited by several factors. Firstly, this study covers most regions of China, due to the differences in technology, equipment, and practices of clinical microbiology laboratories in different regions, the accuracy of bacterial detection and drug sensitivity tests may vary. Second, this study is a literature review and all of the studies were retrospective studies. The integrity and homogeneity of the data are not guaranteed, which may affect the reliability of the results. Despite these limitations, these data provide valuable information on pathogenic bacteria infection and the antibiotic sensitivity of DFUs, which could instruct the initial choice of antibiotics empirically.

## Conclusion

Different regions in China have different pathogen-spectrums on the DFU with a polymicrobial nature. S. aureus, Escherichia coli. and P. aeruginosa were isolated predominantly. GP bacteria were highly sensitive to vancomycin, linezolamide, and teicoranin, and GN bacteria were more susceptible to imipenem, meropenem, piperacillin/tazobactam, and amikacin. Antimicrobial resistance and MDR bacterial infections posed a great challenge to therapy.

## Data Availability Statement

The original contributions presented in the study are included in the article/**Supplementary Material**. Further inquiries can be directed to the corresponding author.

## Author Contributions

FD searched the literature, extracted the data, and drafted this manuscript. JM, HG, RB, PZ, and YR searched the literature. YG, DC, and XR guided the implementation. CW designed the scheme, drafted the manuscript, and revised this draft. All authors reviewed and approved this manuscript.

## Funding

This study was supported by the Science and Technology Bureau of Sichuan Province (Grant No. 2018JY0608).

## Conflict of Interest

The authors declare that the research was conducted in the absence of any commercial or financial relationships that could be construed as a potential conflict of interest.

## Publisher’s Note

All claims expressed in this article are solely those of the authors and do not necessarily represent those of their affiliated organizations, or those of the publisher, the editors and the reviewers. Any product that may be evaluated in this article, or claim that may be made by its manufacturer, is not guaranteed or endorsed by the publisher.

## References

[1] Zhang P Lu J Jing Y Tang S Zhu D Bi Y . Global Epidemiology of Diabetic Foot Ulceration: A Systematic Review and Meta-Analysis (Dagger). Ann Med (2017) 49:106–16. doi: 10.1080/07853890.2016.1231932 27585063

[2] Hurlow JJ Humphreys GJ Bowling FL McBain AJ . Diabetic Foot Infection: A Critical Complication. Int Wound J (2018) 15:814–21. doi: 10.1111/iwj.12932 PMC7949853 29808598

[3] Lipsky BA Berendt AR Cornia PB Pile JC Peters EJ Armstrong DG . Infectious Diseases Society of America Clinical Practice Guideline for the Diagnosis and Treatment of Diabetic Foot Infections. Clin Infect Dis (2012) 54 12:e132-73. doi: 10.1093/cid/cis346 22619242

[4] Peters EJ Lipsky BA . Diagnosis and Management of Infection in the Diabetic Foot. Med Clin North Am (2013) 97:911–46. doi: 10.1016/j.mcna.2013.04.005 23992901

[5] Boyanova L Mitov I . Antibiotic Resistance Rates in Causative Agents of Infections in Diabetic Patients: Rising Concerns. Expert Rev Anti Infect Ther (2013) 11:411–20. doi: 10.1586/eri.13.19 23566150

[6] Bansal E Garg A Bhatia S Attri AK Chander J . Spectrum of Microbial Flora in Diabetic Foot Ulcers. Indian J Pathol Microbiol (2008) 51:204–8. doi: 10.4103/0377-4929.41685 18603682

[7] Moulik PK Mtonga R Gill GV . Amputation and Mortality in New-Onset Diabetic Foot Ulcers Stratified by Etiology. Diabetes Care (2003) 26:491–4. doi: 10.2337/diacare.26.2.491 12547887

[8] Yazdanpanah L Shahbazian H Nazari I Hesam S Ahmadi F Cheraghian B . Risk Factors Associated With Diabetic Foot Ulcer-Free Survival in Patients With Diabetes. Diabetes Metab Syndr (2018) 12:1039–43. doi: 10.1016/j.dsx.2018.06.020 30168426

[9] Miyan Z Fawwad A Sabir R Basit A . Microbiological Pattern of Diabetic Foot Infections at a Tertiary Care Center in a Developing Country. J Pak Med Assoc (2017) 67:665–9. 28507348

[10] Sekhar S Vyas N Unnikrishnan M Rodrigues G Mukhopadhyay C . Antimicrobial Susceptibility Pattern in Diabetic Foot Ulcer: A Pilot Study. Ann Med Health Sci Res (2014) 4:742–5. doi: 10.4103/2141-9248.141541 PMC4199167 25328786

[11] Murali TS Kavitha S Spoorthi J Bhat DV Prasad ASB Upton Z . Characteristics of Microbial Drug Resistance and Its Correlates in Chronic Diabetic Foot Ulcer Infections. J Med Microbiol (2014) 63:1377–85. doi: 10.1099/jmm.0.076034-0 25038136

[12] Reveles KR Duhon BM Moore RJ Hand EO Howell CK . Epidemiology of Methicillin-Resistant Staphylococcus Aureus Diabetic Foot Infections in a Large Academic Hospital: Implications for Antimicrobial Stewardship. PloS One (2016) 11:e0161658. doi: 10.1371/journal.pone.0161658 27556897 PMC4996514

[13] Tascini C Piaggesi A Tagliaferri E Iacopi E Fondelli S Tedeschi A . Microbiology at First Visit of Moderate-to-Severe Diabetic Foot Infection With Antimicrobial Activity and a Survey of Quinolone Monotherapy. Diabetes Res Clin Pract (2011) 94:133–9. doi: 10.1016/j.diabres.2011.07.017 21824673

[14] Aamir AH Nasir A Jadoon MZ Mehmood K Ali SS . Diabetic Foot Infections and Their Management in a Tertiary Care Hospital. J Ayub Med Coll Abbottabad (2011) 23:58–62. 22830148

[15] Malecki R Rosinski K Adamiec R . Etiological Factors of Infections in Diabetic Foot Syndrome - Attempt to Define Optimal Empirical Therapy. Adv Clin Exp Med (2014) 23:39–48. doi: 10.17219/acem/37020 24596002

[16] Wu WX Liu D Wang YW Wang C Yang C Liu XZ . Empirical Antibiotic Treatment in Diabetic Foot Infection: A Study Focusing on the Culture and Antibiotic Sensitivity in a Population From Southern China. Int J Low Extrem Wounds (2017) 16:173–82. doi: 10.1177/1534734617725410 28836481

[17] Hatipoglu M Mutluoglu M Turhan V Uzun G Lipsky BA Turk-Day Study G . Causative Pathogens and Antibiotic Resistance in Diabetic Foot Infections: A Prospective Multi-Center Study. J Diabetes Complications (2016) 30:910–6. doi: 10.1016/j.jdiacomp.2016.02.013 26965794

[18] Mashaly M Kheir MAE Ibrahim M Khafagy W . Aerobic Bacteria Isolated From Diabetic Foot Ulcers of Egyptian Patients: Types, Antibiotic Susceptibility Pattern and Risk Factors Associated With Multidrug-Resistant Organisms. Germs (2021) 11:570–82. doi: 10.18683/germs.2021.1292 PMC8789360 35096674

[19] Banu A Noorul Hassan MM Rajkumar J Srinivasa S . Spectrum of Bacteria Associated With Diabetic Foot Ulcer and Biofilm Formation: A Prospective Study. Australas Med J (2015) 8:280–5. doi: 10.4066/AMJ.2015.2422 PMC4592943 26464584

[20] Dorr S Freier F Schlecht M Lobmann R . Bacterial Diversity and Inflammatory Response at First-Time Visit in Younger and Older Individuals With Diabetic Foot Infection (DFI). Acta Diabetol (2021) 58:181–9. doi: 10.1007/s00592-020-01587-5 32944830

[21] Cervantes-Garcia E Garcia-Gonzalez R Resendiz-Albor A Salazar-Schettino PM . Infections of Diabetic Foot Ulcers With Methicillin-Resistant Staphylococcus Aureus. Int J Low Extrem Wounds (2015) 14:44–9. doi: 10.1177/1534734614564053 25573977

[22] Commons RJ Robinson CH Gawler D Davis JS Price RN . High Burden of Diabetic Foot Infections in the Top End of Australia: An Emerging Health Crisis (DEFINE Study). Diabetes Res Clin Pract (2015) 110:147–57. doi: 10.1016/j.diabres.2015.09.016 PMC4684095 26453263

[23] Shettigar K Murali TS . Virulence Factors and Clonal Diversity of Staphylococcus Aureus in Colonization and Wound Infection With Emphasis on Diabetic Foot Infection. Eur J Clin Microbiol Infect Dis (2020) 39:2235–46. doi: 10.1007/s10096-020-03984-8 PMC7669779 32683595

[24] Al Benwan K Al Mulla A Rotimi VO . A Study of the Microbiology of Diabetic Foot Infections in a Teaching Hospital in Kuwait. J Infect Public Health (2012) 5:1–8. doi: 10.1016/j.jiph.2011.07.004 22341838

[25] Kurup R Ansari AA . A Study to Identify Bacteriological Profile and Other Risk Factors Among Diabetic and Non-Diabetic Foot Ulcer Patients in a Guyanese Hospital Setting. Diabetes Metab Syndr (2019) 13:1871–6. doi: 10.1016/j.dsx.2019.04.024 31235108

[26] Raja NS . Microbiology of Diabetic Foot Infections in a Teaching Hospital in Malaysia: A Retrospective Study of 194 Cases. J Microbiol Immunol Infect (2007) 40:39–44. 17332905

[27] Stein GE Schooley S Peloquin CA Missavage A Havlichek DH . Linezolid Tissue Penetration and Serum Activity Against Strains of Methicillin-Resistant Staphylococcus Aureus With Reduced Vancomycin Susceptibility in Diabetic Patients With Foot Infections. J Antimicrob Chemother (2007) 60:819–23. doi: 10.1093/jac/dkm271 17673476

[28] Anvarinejad M Pouladfar G Japoni A Bolandparvaz S Satiary Z Abbasi P . Isolation and Antibiotic Susceptibility of the Microorganisms Isolated From Diabetic Foot Infections in Nemazee Hospital, Southern Iran. J Pathog (2015) 2015:328796. doi: 10.1155/2015/328796 26843987 PMC4710915

[29] Stacey HJ Clements CS Welburn SC Jones JD . The Prevalence of Methicillin-Resistant Staphylococcus Aureus Among Diabetic Patients: A Meta-Analysis. Acta Diabetol (2019) 56:907–21. doi: 10.1007/s00592-019-01301-0 PMC6597605 30955124

[30] Uckay I Gariani K Pataky Z Lipsky BA . Diabetic Foot Infections: State-of-the-Art. Diabetes Obes Metab (2014) 16:305–16. doi: 10.1111/dom.12190 23911085

[31] Livermore DM . The Need for New Antibiotics. Clin Microbiol Infect (2004) 10:1–9. doi: 10.1111/j.1465-0691.2004.1004.x 15522034

[32] Sun S Wang C Chen D Cen S Lv X Wen X . Combating Superbug Without Antibiotic on a Postamputation Wound in a Patient With Diabetic Foot. Int J Low Extrem Wounds (2016) 15:74–7. doi: 10.1177/1534734615595736 26238676

[33] Pouget C Dunyach-Remy C Pantel A Schuldiner S Sotto A Lavigne JP . Biofilms in Diabetic Foot Ulcers: Significance and Clinical Relevance. Microorganisms (2020) 8:1580. doi: 10.3390/microorganisms8101580 PMC7602394 33066595

[34] Steffy K Shanthi G Maroky AS Selvakumar S . Potential Bactericidal Activity of S. Nux-Vomica-ZnO Nanocomposite Against Multidrug-Resistant Bacterial Pathogens and Wound-Healing Properties. J Trace Elem Med Biol (2018) 50:229–39. doi: 10.1016/j.jtemb.2018.07.009 30262284

[35] Abdulrazak A Bitar ZI Al-Shamali AA Mobasher LA . Bacteriological Study of Diabetic Foot Infections. J Diabetes Compl (2005) 19:138–41. doi: 10.1016/j.jdiacomp.2004.06.001 15866058

[36] Mlinaric Missoni E Vukelic M de Soy D Belicza M Vazic Babic V Missoni E . Fungal Infection in Diabetic Foot Ulcers. Diabetes Med (2005) 22:1124–5. doi: 10.1111/j.1464-5491.2005.01611.x 16026387

[37] Kalan L Loesche M Hodkinson BP Heilmann K Ruthel G Gardner SE . Redefining the Chronic-Wound Microbiome: Fungal Communities Are Prevalent, Dynamic, and Associated With Delayed Healing. mBio (2016) 7:e01058-16. doi: 10.1128/mBio.01058-16 27601572 PMC5013295

[38] Ramakant P Verma AK Misra R Prasad KN Chand G Mishra A . Changing Microbiological Profile of Pathogenic Bacteria in Diabetic Foot Infections: Time for a Rethink on Which Empirical Therapy to Choose? Diabetologia (2011) 54:58–64. doi: 10.1007/s00125-010-1893-7 20835702

[39] Hitam SAS Hassan SA Maning N . The Significant Association Between Polymicrobial Diabetic Foot Infection and Its Severity and Outcomes. Malays J Med Sci (2019) 26:107–14. doi: 10.21315/mjms2019.26.1.10 PMC6419864 30914898

[40] Citron DM Goldstein EJ Merriam CV Lipsky BA Abramson MA . Bacteriology of Moderate-to-Severe Diabetic Foot Infections and *In Vitro* Activity of Antimicrobial Agents. J Clin Microbiol (2007) 45:2819–28. doi: 10.1128/JCM.00551-07 PMC2045270 17609322

[41] von Eiff C Peters G Heilmann C . Pathogenesis of Infections Due to Coagulase-Negative Staphylococci. Lancet Infect Dis (2002) 2:677–85. doi: 10.1016/s1473-3099(02)00438-3 12409048

[42] Castellanos N Nakanouchi J Yuzen DI Fung S Fernandez JS Barberis C . A Study on Acinetobacter Baumannii and Staphylococcus Aureus Strains Recovered From the Same Infection Site of a Diabetic Patient. Curr Microbiol (2019) 76:842–7. doi: 10.1007/s00284-019-01696-7 PMC6556059 31053906

[43] Bader MS . Diabetic Foot Infection. Am Fam Physician (2008) 78:71–9. 18649613

