# Supplementary material for: Microbial Infection and Antibiotic Susceptibility of Diabetic Foot Ulcer in China: Literature Review
Source: Front Endocrinol (Lausanne). 2022 May 19;13:881659. doi: 10.3389/fendo.2022.881659 (PMC9161694; doi:10.3389/fendo.2022.881659)
Supplement: Supplementary file 1 [file DataSheet_1.docx]

Sixty-three clinical articles included in this study

1. 王彬.38例糖尿病足感染患者病原菌特点及药敏回顾分析[J].糖尿病新世界,2015(03):7-8.DOI:10.16658/j.cnki.1672-4062.2015.03.104.
2. 姚兰,安民民.95例糖尿病足部感染患者病原菌分布及耐药性分析[J].皖南医学院学报,2015,34(05):464-468.
3. 陈燕,陈卫红,何亮军.125例糖尿病足感染患者病原菌分布及耐药性分析[J].临床与病理杂志,2015,35(06):1004-1009.
4. 刘敏洁,李高申,薛现军,郑勇.糖尿病患者感染的病原菌分布与耐药性研究[J].中华医院感染学杂志,2015,25(07):1507-1508+1529.
5. 叶满.糖尿病足病原菌的耐药分析[J].右江民族医学院学报,2015,37(05):700-702.
6. 张洪,张文广,蔡文智,郭红卫,王位琼,许振华.糖尿病足感染病原菌分布、药敏及Silverloy抗菌效果[J].广东医学,2015,36(16):2520-2522.DOI:10.13820/j.cnki.gdyx.2015.16.027.
7. 刘阳,张婉,黄超,胡玲玲.糖尿病足感染病原菌分析[J].河北联合大学学报(医学版),2015,17(06):185-187.DOI:10.19539/j.cnki.2095-2694.2015.06.007.
8. 周滇,王雯,虞俊杰,吕国忠.糖尿病足感染的病原菌及耐药性分析[J].临床荟萃,2015,30(03):304-307.
9. 陈义阳,郭世辉,黄松,黄振兴,梁杏欢,秦映芬,等.糖尿病足感染的病原菌及其耐药性分析[J].广西医科大学学报,2015,32(02):238-240.DOI:10.16190/j.cnki.45-1211/r.2015.02.022.
10. 饶小胖,揭小鸣,宋振华.糖尿病足溃疡创面特点及主要细菌分布的研究[J].微生物与感染,2015,10(06):359-364.
11. 邱平,梅希,唐明薇,廖戮缪,卢迪,杨惠岚,等.88例糖尿病足感染的病原菌分布和药敏分析[J].吉林医学,2016,37(02):290-293.
12. 刁斌斌.糖尿病足部感染患者病原菌检验与耐药性分析[J].糖尿病新世界,2016,19(21):31-32.DOI:10.16658/j.cnki.1672-4062.2016.21.031.
13. 何灵杰,殷礼君,张秀薇,陈伟坤,李金德.糖尿病足患者病原菌特点及药敏分析[J].广东医学,2016,37(S1):200-201.DOI:10.13820/j.cnki.gdyx.2016.s1.088.
14. 林鹭平,罗芳涛,张劼.糖尿病足56例病原菌培养结果分析[J].山西医药杂志,2016,45(08):962-964.
15. 刘颖芳.糖尿病足复发感染病原菌的分布及耐药性分析[J].中国微生态学杂志,2016,28(11):1305-1308.DOI:10.13381/j.cnki.cjm.201611018.
16. 陈芳,李素梅,王炜,宫阿娟,胡俊程.糖尿病足患者足部创面感染的病原菌分布及药敏结果分析[J].山东医药,2016,56(30):49-51.
17. 楼百层,梅健慧,骆京京,胡娅娜,晏程远.糖尿病足患者感染病原菌分布与耐药性分析[J].中华医院感染学杂志,2016,26(14):3232-3234.
18. 王艳,尹丽,钱灿,尹翰林,陈雅雯,樊莲莲,等.145例糖尿病足创面病原菌特点及药敏分析[C]. 2016:386-386.
19. 高家林,张魏,刘春声,朱玉,夏礼斌,陈月平,等.皖南地区898例糖尿病足病及烧烫伤创面分泌物培养病原菌及耐药分析[J].中国临床药理学与治疗学,2016,21(07):796-801.
20. 赵亮,程庆丰,朱深银,向翼,李欣宇,邱峰,等.基于Wagner分级糖尿病足感染的病原菌分布及药敏分析[J].重庆医科大学学报,2016,41(11):1105-1109.DOI:10.13406/j.cnki.cyxb.001051.
21. 江浩.2型糖尿病患者糖尿病足复发感染的病原菌特点及危险因素分析[J].广东医科大学学报,2017,35(05):474-477.
22. 王亮萍,陈闽.糖尿病足感染病原菌分布特点及预后分析[J].实用糖尿病杂志,2017,13(03):32-34.
23. 周秀芳.糖尿病足感染的细菌谱变迁与抗感染治疗[J].大医生,2017,2(Z1):52-54.DOI:10.19604/j.cnki.dys.2017.z1.019.
24. 乐忠宏,汤晓姣,崔婷婷,张洁,王姣.糖尿病足感染患者病原学特点及耐药性分析[J].中华医院感染学杂志,2017,27(03):590-592+601.
25. 赵文霞,李剑芳,邱凯锋.糖尿病足感染患者致病菌分布及药物敏感试验结果分析[J].中国医院用药评价与分析,2017,17(10):1425-1427+1430.DOI:10.14009/j.issn.1672-2124.2017.10.045.
26. 何杏仪,黄景胜,张莉.糖尿病足合并感染患者病原菌分布及耐药性变迁[J].今日药学,2017,27(11):767-770.
27. 李珍,区岛良,李文霞,李美花,赵汉儒,杨祚明.糖尿病足患者感染病原学特点及药敏分析[J].中华医院感染学杂志,2017,27(20):4653-4656+4660.
28. 谢光云,吴世木,刘爽,张翠,李升钦.糖尿病足患者伤口感染的病原菌分布及药敏结果分析[J].中华医院感染学杂志,2017,27(17):3897-3900.
29. 黄婵娟,史金转,罗德钦,林在兰,黄育姑.糖尿病足患者足部创面感染病原学特点分析[J].中国地方病防治杂志,2017,32(04):447.
30. 颜新星.糖尿病足部溃疡感染病原菌分布及药物敏感性分析[J].双足与保健,2018,27(23):36-37.DOI:10.19589/j.cnki.issn1004-6569.2018.23.036.
31. 林丽玲,邱秀兰,翁月萍,陈夏容,吴逸海.糖尿病足部溃疡患者感染病原菌分布与临床治疗评价[J].糖尿病新世界,2018,21(15):176-177.DOI:10.16658/j.cnki.1672-4062.2018.15.176.
32. 刘宇彪,周花玩.糖尿病足患者感染病原菌及药敏分析[J].现代医学与健康研究电子杂志,2018,2(17):46+48.
33. 肖贵宝,陶世冰,陈刚,宋敏.糖尿病足患者感染病原菌分布特点及对抗菌药物的敏感性分析[J].中华医院感染学杂志,2018,28(07):1033-1036.
34. 李琳,皮银珍,胡金伟,胡丽.糖尿病足患者感染病原菌特点及耐药性分析[J].临床合理用药杂志,2018,11(17):5-6.DOI:10.15887/j.cnki.13-1389/r.2018.17.003.
35. 王广辉,吴雁翔.住院糖尿病足患者足部溃疡分泌物病原菌及药敏分析[J].中国民康医学,2018,30(14):96-97.
36. 陈丽华,杨婧,伍勇.糖尿病足患者足分泌物分离菌分布及多重耐药影响因素分析[J].中国抗生素杂志,2018,43(10):1286-1290.DOI:10.13461/j.cnki.cja.006408.
37. 王赐玉,黄昭瑄,黄昭穗,栾丽丽,何艺芬.糖尿病患者足部溃疡感染的病原菌分布及药敏性分析[J].现代生物医学进展,2018,18(17):3327-3331.DOI:10.13241/j.cnki.pmb.2018.17.027.
38. 张朝光,戴黎明,杨夏.糖尿病足患者下肢血管病变与细菌感染的关系研究[J].中华医院感染学杂志,2018,28(01):51-54+61.
39. 徐晓俊.糖尿病足溃疡感染与病原菌种及敏感抗生素的关系研究[J].全科口腔医学电子杂志,2018,5(36):95-96+101.DOI:10.16269/j.cnki.cn11-9337/r.2018.36.065.
40. 杜鸣,刘佳霖,许鑫,陈敏,廖亚玲,胡光煦,等.糖尿病足溃疡与感染患者病原菌分布与合理抗菌药物的选用分析[J].现代预防医学,2018,45(04):737-741.
41. 崔金国,刘俊杰,孙景生.2016—2017年天津市宝坻区人民医院糖尿病足患者病原菌分布及药敏性分析[J].现代药物与临床,2019,34(02):554-557.
42. 简丽,赵启全,何阳杰,贾蓓,程庆丰.2015～2017年我院基于Wagner分级糖尿病足患者细菌耐药检测结果分析[J].临床内科杂志,2019,36(04):261-263.
43. 甘美舍,陈秀林,杨斌,潘美时,黄丽娜,劳志醒,等. 2型糖尿病患者糖尿病足感染的细菌学分析[J]. 特别健康,2019(35):46-47. DOI:10.3969/j.issn.2095-6851.2019.35.066.
44. 毕然然,许玉华,王绵,刘瑞红,陈枫,牟荣菲,等.住院患者中糖尿病足感染病原菌及相关因素分析[J].中华糖尿病杂志,2019(12):782-787.
45. 王帅,吕大伦,李敏,端龙胜,丁伟,王合丽,等.52例糖尿病足患者创面微生物分布及药物敏感分析[J].皖南医学院学报,2019,38(06):567-571.
46. 曹绮雯,杨群峰,万杰君.77例糖尿病足部溃疡患者分泌物培养及对抗菌药物耐药性分析[J].广州医科大学学报,2019,47(06):64-67.
47. 杨正海,王章凤.133例糖尿病足感染病原菌分布及耐药性分析[J].双足与保健,2019,28(18):46-47+49.DOI:10.19589/j.cnki.issn1004-6569.2019.18.046.
48. 杨雪梅,林珊珊,郭丽敏.150例糖尿病足感染患者细菌分布及药敏反应特点[J].山东医药,2019,59(25):76-78.
49. 刘春林,徐波,陈弟,张莉,王飞英,徐红云,等.感染性糖尿病足病原菌分布及耐药性分析[J].检验医学,2019,34(06):513-517.
50. 林久龙.糖尿病足部感染患者病原菌检验与耐药性分析[J].双足与保健,2019,28(23):18-20.DOI:10.19589/j.cnki.issn1004-6569.2019.23.018.
51. 赵平,陈亮,解泽强,菅记涌,张曼.糖尿病足感染病原菌分布和耐药特点研究[J].国际检验医学杂志,2019,40(18):2195-2199.
52. 王小芳,陈军,陈宗涛.糖尿病足感染病原菌构成及耐药情况分析[J].中国病原生物学杂志,2019,14(04):477-481.DOI:10.13350/j.cjpb.190423.
53. 翟伟伟.糖尿病足部感染患者病原菌检验与耐药性分析[J].双足与保健,2019,28(16):5-6.DOI:10.19589/j.cnki.issn1004-6569.2019.16.005.
54. 邱潇.糖尿病足感染患者的病原菌特点及药敏试验研究[J].双足与保健,2019,28(24):21-22+25.DOI:10.19589/j.cnki.issn1004-6569.2019.24.021.
55. 王瑶,梁静.糖尿病足合并周围血管病变患者的病原菌及药敏分析研究[J].世界最新医学信息文摘,2019,19(63):234-235.DOI:10.19613/j.cnki.1671-3141.2019.63.130.
56. 刘薇,张如意,刘剑烽,冉建民,赖美铮,谢彬.糖尿病足溃疡面病原菌与影响因素分析[J].实用医学杂志,2019,35(15):2443-2446.
57. 刘海楠,苏莎莎,刘伟,张彦忠.糖尿病足下肢血管病变程度对细菌感染病原菌谱及耐药性影响分析[J].中华医院感染学杂志,2019,29(10):1504-1508.
58. 穆琪,钱超,常琪,余建华,张子烨.糖尿病足溃疡患者病原菌感染特征分析[J].东南国防医药,2019,21(05):460-465.
59. 谢树永,牛敏,申金付,罗莉,章秋.糖尿病足复发感染不同严重程度分级中病原菌分布特点研究[J].中国全科医学,2020,23(08):919-922.
60. 王红梅,石锋,吴永新,马苑霞.基于Wagner分级糖尿病足感染的病原菌分布及药敏分析[J].包头医学院学报,2019,35(11):25-27.DOI:10.16833/j.cnki.jbmc.2019.11.009.
61. Liu L, Li Z, Liu X, Guo S, Guo L, Liu X. Bacterial distribution, changes of drug susceptibility and clinical characteristics in patients with diabetic foot infection. Exp Ther Med. 2018;16(4):3094-3098. doi:10.3892/etm.2018.6530
62. Li X, Qi X, Yuan G, et al. Microbiological profile and clinical characteristics of diabetic foot infection in northern China: a retrospective multicentre survey in the Beijing area. J Med Microbiol. 2018;67(2):160-168. doi:10.1099/jmm.0.000658
63. Wu M, Pan H, Leng W, Lei X, Chen L, Liang Z. Distribution of Microbes and Drug Susceptibility in Patients with Diabetic Foot Infections in Southwest China. J Diabetes Res. 2018;2018:9817308. Published 2018 Aug 5. doi:10.1155/2018/9817308

1.Wang B. Retrospective Analysis of the Pathogenic Characteristics of 38 Cases with Diabetic Foot Infection and the Drug sensitivity. DIABETES NEW WORLD (2015) (03):7-8. doi:10.16658/j.cnki. 1672-4062.2015.03.104

2.Yao L, An MM. Distribution and resistance surveillance of pathogens in 95 cases of diabetic foot infections. J of Wannan Medical College (2015) 34:464-8.

3.Chen Y, Chen WH, He LJ. Distribution and resistance surveillance of pathogens causing diabetic foot infections in 125 cases. J Clin Pathol Res (2015) 35:1004-9.

4.Liu MJ, Li GS, Xue XJ, Zheng Y. Distribution of infected diabetic patients and drug resistance of pathogenic bacteria. Chin J Nosocomiol (2015) 25:1507-8+29.

5.Ye M. The drug-resistance analysis of diabetic foot pathogen. Journal of Youjiang Medical University for Nationnalities (2015) 37:700-2.

6.Zhang H, Zhang WG, Cai WZ, Guo HW, Wang WQ, Xu ZH. Distribution, drug sensitivity and antibacterial effect of Silverloy on diabetic foot infections. Guangdong Medical Journal (2015) 36:2520-2. doi:10.13820/j.cnki.gdyx.2015.16.027

7.Liu Y, Zhang W, Huang C, Hu LL. Analysis of pathogenic bacteria in diabetic foot infection. Journal of Hebei United University (Health Sciences) (2015) 17:185-7. doi:10.19539/j.cnki.2095-2694.2015.06.007

8.Zhou D, Wang W, Yu JJ, Lv GZ. Distribution and drug resistance analysis of pathogenic bacteria in diabetic foot infection. Clinical Focus (2015) 30:304-7.

9.Chen YY, Guo SH, Huang S, Huang ZX, Liang XH, Qin YF. Pathogenic bacteria and drug resistance of diabetic foot infection. Journal of Guangxi Medical University (2015) 32:238-40. doi:10.16190/j.cnki.45-1211/r.2015.02.022

10.Rao XP, Jie XM, Song ZH. A study of diabetic foot ulcer: Clinical characteristics and bacterial distribution. Journal of Microbes and Infections (2015) 10:359-64.

11.Qiu P, Mei X, Tang MW, Liao LM, Lu D, Yang HL, et al. Distribution and antibiotic susceptibility of pathogens isolated from 88 patients with diabetic foot infec- tion in northern district of Chengdu. Jilin Medical Journal (2016) 37:290-3.

12.Diao BB. Pathogen detection and drug resistance in patients with diabetic foot infection. DIABETES NEW WORLD (2016) 19:31-2. doi:10.16658/j.cnki.1672-4062.2016.21.031

13.He LJ, Yin LJ, Zhang XW, Chen WK, Li JD. Pathogen characteristics and drug sensitivity analysis of diabetic foot patients. Guangdong Medical Journal (2016) 37:200-1. doi:10.13820/j.cnki.gdyx.2016.s1.088

14.Lin LP, Luo FT, Zhang J. Analysis of pathogenic bacteria culture in 56 patients with diabetic foot. Shanxi Med J (2016) 45:962-4.

15.Liu YF. Antibiotic-resistance and distribution of pathogenic bacteria of recurrent diabetic foot infections. Chin J Microecol (2016) 28:1305-8. doi:10.13381/j.cnki.cjm.201611018

16.Chen F, Li SM, Wang W, Gong AJ, Hu JC. Pathogenic bacteria distribution and drug sensitivity analysis of foot wound infection in diabetic foot patients. Shandong Medical Journal (2016) 56:49-51.

17.Lou BC. Distribution and drug resistance of pathogens causing infections in diabetic foot patients. Chin J Nosocomiol (2016) 26:3232-4.

18.Wang Y, Yin L, Qian C, Yin HL, Chen YW, Fan LL, et al. Pathogenic bacteria and drug sensitivity analysis of 145 patients with diabetic foot wounds. Chinese Journal of Hospital Pharmacy (2016):386.

19.Gao JL, Zhang W, Liu CS, Zhu Y, Xia LB, Chen YP, et al. Pathogenic bacteria and drug resistance analysis of 898 cases of diabetic foot and scald wound secretion in southern Anhui. Chin J Clin Pharm Therap (2016) 21:796-801.

20.Zhao L, Cheng QF, Zhu SY, Xiang Y, Li XY, Qiu F, et al. Distribution and antibiotic susceptibility of pathogens in diabetic foot infection based on Wagner grade. Journal of Chongqing Medical University (2016) 41:1105-9. doi:10.13406/j.cnki.cyxb.001051

21.Jiang H. Analysis on the characteristics and risk factors of the pathogenic bacteria of recurrent diabetic foot infection in Type 2 diabetic mellitus patients. JOURNAL OF GUANGDONG MEDICAL UNIVERSITY (2017) 35:474-7.

22.Wang LP, Chen M. Distribution characteristics of pathogenic bacteria and prognostic analysis in patients with diabetic foot infection. JOUANAL OF PRACTICAL DIABETOLOGY (2017) 13:32-4.

23.Zhou XF. Changes of Bacterial Spectrum and Anti in Fection Treatment of Diabetic Foot Infection. Preventive Medicine (2017) 2:52-4. doi:10.19604/j.cnki.dys.2017.z1.019

24.Le ZH, Tang XJ, Cui TT, Zhang J, Wang J. Pathogenic bacteriological characteristics and drug resistance of patients with diabetic foot. Chin J Nosocomiol (2017) 27:590-2+601.

25.Zhao WX, Li JF, Qiu KF. Analysis on Distribution of Pathogenic Bacteria and Ｒesults of Drug Sensitivity Tests Among Patients with Diabetic Foot Infection. Evaluation and analysis of drug-use in hospitals of China (2017) 17:1425-7+30. doi: 10.14009/j.issn.1672-2124.2017.10.045

26.He XY, Huang JS, Zhang L. Trend in Distribution and Antibacterial Resistance of Pathogens in Diabetic Foot Complicated with Infection. Pharmacy Today (2017) 27:767-70.

27.Li Z, Ou DL, Li WX, Li MH, Zhao HR, Yang ZM. Pathogenic characteristics and drug sensitivity analysis of infections in patients with diabetic foot. Chin J Nosocomiol (2017) 27:4653-6+60.

28.Xie GY, Wu SM, Liu S, Zhang C, Li SQ. Distribution and drug suscepbility of pathogens causing wound infections in diabetic foot patients. Chin J Nosocomiol (2017) 27:3897-900.

29.Huang CJ, Shi JZ,Luo DQ, Lin ZL,Huang YG. Etiological characteristics of foot wound infection in diabetic foot patients. Chin J Ctrl Endem Dis (2017) 32:447.

30.Yan XX. Distribution and drug sensitivity analysis of pathogens causing infections in diabetic foot patients. China Reflexolocy (2018) 27:36-7. doi:10.19589/j.cnki.issn1004-6569.2018.23.036

31.Lin LL, Qiu XL, Weng YP, Chen XR, Wu YH. Distribution of pathogenic bacteria and clinical evaluation in patients with diabetic foot ulcer. DIABETES NEW WORLD (2018) 21:176-7. doi:10.16658/j.cnki.1672-4062.2018.15.176

32.Liu YB, Zhou HW. Analysis of pathogenic bacteria and drug sensitivity in diabetic foot patients. Modern Medicine and Health Research (2018) 2:46+8.

33.Xiao GB, Tao SB, Chen G, Song M. Distribution of pathogenic bacteria in diabetic foot infection and their sensitivity to antibiotics. Chin J Nosocomiol (2018) 28:1033-6.

34.Li L, Pi YZ, Hu JW, Hu L. Characteristics of pathogenic bacteria and analysis of drug resistance in diabetic foot patients. Chin J of Clinical Rational Drug Use (2018) 11:5-6. doi:10.15887/j.cnki.13-1389/r.2018.17.003

35.Wang GH, Wu YX. Pathogenic bacteria and drug sensitivity analysis of foot ulcer secretion in hospitalized diabetic foot patients. Medical Journal of Chinese People＇s Health (2018) 30:96-7.

36.Chen LH, Yang J, Wu Y. An analysis of the distribution of pathogenic bacteria in patients with diabetic foot and factors related to drug resistance. Chin J Antibio (2018) 43:1286-90. doi:10.13461/j.cnki.cja.006408

37.Wang CY, Huang ZX, Huang ZS, Luan LL, He YF. Distribution of Pathogens and Analysis of Drug Susceptibility on Patients with Diabetic Foot Ulcer Infection. Progress in Modern Biomedicine (2018) 18:3327-31. doi:10.13241/j.cnki.pmb.2018.17.027

38.Zhang CG, Dai LM, Yang X. Relationship between lower extremity vascular lesions and bacterial infection in patients with diabetic foot. Chin J Nosocomiol (2018) 28:51-4+61.

39.Xu XJ. Relationship between infection of diabetic foot ulcers and pathogenic bacteria and sensitive antibiotics. General Journal Of Stomatology (2018) 5:95-6+101. doi:10.16269/j.cnki.cn11-9337/r.2018.36.065

40.Du M, Liu JL, Xu X, Chen M, Liao YL, Hu GX, et al. Analysis on distribution and drug resistance of pathogens causing diabetic foot ulcer and infections to support clinical rational drug use. Modern Preventive Medicine (2018) 45:737-41.

41.Cui JG, Liu JJ, Sun JS. Analysis on distribution and drug resistance of pathogens of patients with diabetic foot infections in Tianjin Baodi People’s Hospital from 2016 to 2017. Drugs & Clinic (2019) 34:554-7.

42.Jian L, Zhao QQ, He YJ, Jia B, Cheng QF. Analysis of bacterial resistance in diabetic foot patients based on Wagner classification from 2015 to 2017. J Clin Inter Med (2019) 36:261-3.

43.Gan MS, Chen XL, Yang B, Pan MS, Huang LN, Lao ZX, et al. Bacteriological analysis of diabetic foot infection in patients with type 2 diabetes mellitus. Special Health (2019) (35):46-7. doi:10.3969/j.issn.2095-6851.2019.35.066

44.Bi RR, Xu YH, Wang M, Liu RH, Chen F, Mou RF, et al. Pathogenic bacteria and related factors of diabetic foot infection in hospitalized patients. Chin J Diabetes Mellitus (2019) (12):782-7.

45.Wang S, Lv DL, Li M, Duan LS, Ding W, Wang HL, et al. Microbial distribution and drug sensitivity in diabetic foot wounds: Retrospective analysis in 52 cases. J of Wannan Medical College (2019) 38:567-71.

46.Cao QW, Yang QF, Wan JJ. Secretion culture and antibiotic resistance in 77 patients with diabetic foot ulcer. ACADEMIC JOURNAL OF GUANGZHOU MEDICAL UNIVERSITY (2019) 47:64-7.

47.Yang ZH, Wang ZF. Distribution and drug resistance of pathogenic bacteria in 133 cases of diabetic foot infection. China Reflexolocy (2019) 28:46-7+9. doi:10.19589/j.cnki.issn1004-6569.2019.18.046

48.Yang HM, Lin SS, Guo LM. Characteristics of bacterial distribution and drug sensitivity in 150 patients with diabetic foot infection. Shandong Medical Journal (2019) 59:76-8.

49.Liu CL, Xu B, Chen D, Zhang L, Wang FY, Xu HY, et al. Distribution and drug resistance analysis of pathogenic bacteria isolated from infectious patients with diabetic foot. Laboratory Medicine (2019) 34:513-7.

50.Lin JL. Analysis of Pathogens and Drug Resistance in Patients with Diabetic Foot Infection. China Reflexolocy (2019) 28:18-20. doi:10.19589/j.cnki.issn1004-6569.2019.23.018

51.Zhao P, Chen L, Xie ZQ, Jian JY, Zhang M. The microbial profile and antibiotic resistance of bacterial pathogens from diabetic foot infections. Int J Lab Med (2019) 40:2195-9.

52.Wang XF, Chen J, Chen ZT. The distribution of pathogens causing foot infections in diabetics. Journal of Pathogen Biology (2019) 14:477-81. doi:10.13350/j.cjpb.190423

53.Zhai WW. Analysis of Pathogens and Drug Resistance in Patients with Diabetic Foot Infection. China Reflexolocy (2019) 28:5-6. doi:10.19589/j.cnki.issn1004-6569.2019.16.005

54.Qiu X. Study on Pathogenic Bacteria Characteristics and Drug Sensitivity Test in Patients with Diabetic Foot Infection. China Reflexolocy (2019) 28:21-2+5. doi:10.19589/j.cnki.issn1004-6569.2019.24.021

55.Wang Y, Liang J. Analysis of pathogenic bacteria and drug sensitivity in diabetic foot patients with peripheral vascular disease. World Latest Medicine Information (Electronic Version) (2019) 19:234-5.doi:10.19613/j.cnki.1671-3141.2019.63.130

56.Liu W, Zhang RY, Liu JF, Ran JM, Lai MZ, Xie B. Analysis of pathogenic bacteria and influencing factors of diabetic foot ulcer surface. The Journal of Practical Medicine (2019) 35:2443-6.

57.Liu HN, Su SS, Liu W, Zhang YZ. Influence of vascular lesions in lower extremities of diabetic foot patients on spectrum and drug resistance of pathogens causing foot infection. Chin J Nosocomiol (2019) 29:1504-8.

58.Mu Q, Qian C, Chang Q, Yu JH, Zhang ZY. Characteristic analysis of pathogenic bacteria infection in patients with diabetic foot ulcer. Military Medical Journal of Southeast China (2019) 21:460-5.

59.Xie SY, Niu M, Shen JF, Luo L, Zhang Q. Distribution Characteristics of Pathogenic Bacteria in Different Severity Grades of Recurrent Diabetic Foot Infection. Chin Gen Prac (2020) 23:919-22.

60.Wang HM , Shi F, Wu YX, Ma YX. Distribution and drug sensitivity analysis of pathogenic bacteria in diabetic foot infection based on Wagner classification. Journal of Baotou Medical Colleg (2019) 35: 25-7. doi:10.16833/j.cnki.jbmc.2019.11.009

61.Liu L, Li Z, Liu X, Guo S, Guo L, Liu X. Bacterial distribution, changes of drug susceptibility and clinical characteristics in patients with diabetic foot infection. Exp Ther Med. 2018;16(4):3094-3098. doi:10.3892/etm.2018.6530

62.Li X, Qi X, Yuan G, et al. Microbiological profile and clinical characteristics of diabetic foot infection in northern China: a retrospective multicentre survey in the Beijing area. J Med Microbiol. 2018;67(2):160-168. doi:10.1099/jmm.0.000658

63.Wu M, Pan H, Leng W, Lei X, Chen L, Liang Z. Distribution of Microbes and Drug Susceptibility in Patients with Diabetic Foot Infections in Southwest China. J Diabetes Res. 2018;2018:9817308. Published 2018 Aug 5. doi:10.1155/2018/9817308
